# Editorial: The emerging role of endothelial cells in vascular and metabolic disorders; endothelium regeneration and vascular repair is the future for therapeutics

**DOI:** 10.3389/fcell.2024.1512568

**Published:** 2024-11-19

**Authors:** Pratibha Bhalla, Ondine Cleaver

**Affiliations:** ^1^ Department of Immunology, The University of Texas Southwestern Medical Center, Dallas, TX, United States; ^2^ Molecular Biology, The University of Texas Southwestern Medical Center, Dallas, TX, United States

**Keywords:** vascular disease, endothelial cells dysfunction, metabolic disorder, aging, diabetic retinopathy, Alzheimer’s disease

The vasculature plays a critical role in numerous diseases, influencing disease progression, treatment efficacy, and patient outcomes. The term ‘vasculature’ refers to the entire network of blood vessels, comprising arteries, arterioles, veins, venules and capillaries. The circulatory system, including all these vessels, is lined by endothelial cells (ECs). This single-cell-thick layer of ECs, also known as vascular endothelium (VE), serves as a critical interface between circulating blood and the tissues of the body, and recently been referred to as the largest endocrine organ in the body. ECs produce and release various vasoactive factors that help regulate blood flow (nitric oxide (NO), and endothelin-1), blood pressure (angiotensin-converting enzyme, ACE), coagulation (von Willebrand factor, vWF), and fibrinolytic balance (prostacyclin and tissue plasminogen activator, tPA). Activation of ECs changes the expression of certain genes (such as vascular endothelial growth factor A, VEGFA, and angiopoietin-2, ANGPT2), and when excessively activated, these cells can become dysfunctional or promote unnecessary angiogenesis. Endothelial cells (ECs) show abnormal activation or dysfunction in a range of conditions, such as atherosclerosis, diabetes, obesity, neurodegenerative diseases, and liver disorders; however, their precise role and the pathways they influence in regulating metabolic homeostasis remain poorly understood till today (Peng et al., 2021; Pober et al., 2009; Ungvari et al., 2018).

It is essential to understand the roles of vascular factors in normal and diseased vessels for developing targeted therapies and preventive strategies for various health conditions. In the series of articles collected in the Research Topic entitled “The emerging role of endothelial cells in vascular and metabolic disorders; endothelium regeneration and vascular repair is the future for therapeutics” novel insights on vascular mediated disorders and potential therapeutic targets are presented.

The review by the Qiu et al. group discusses about several perioperative factors such as temperature changes, pain, opioid use, and anesthesia that can disrupt the blood-brain barrier (BBB), particularly in older adults or those with neurodegenerative diseases such as Alzheimer’s disease (AD). The collapsing of the blood-brain barrier during the perioperative period is directly linked to cognitive impairment and may elevate the risk of postoperative mortality. The BBB is a complex structure formed by ECs, astrocytes, pericytes, and the extracellular matrix, working together to create a highly selective barrier. Surgery by itself amplifies BBB damage, increasing inflammation and neuroinflammation. In addition, anesthesia also damages BBB by disrupting tight junction, causing endothelial dysfunction. Intestinal imbalance in gut microbiota occurring during the perioperative period is found to enhance BBB damage through neurotransmitters and short-chain fatty acids. Use of probiotics is demonstrated to strengthen tight junctions and reduce BBB permeability, restoring endothelial integrity and improving cognitive outcomes.

Another review by Milani et al. group emphasizes the role of autophagy in vascular cell function, particularly in pericytes, both under physiological and pathological conditions. Pericytes, which ensheathe ECs in most organs and tissues, are known to be essential for vascular integrity. Autophagy in many different cell types, including pericytes, acts as an early resistance mechanism against metabolic disorders. This review article delves into the mechanisms underlying both the protective and detrimental roles of autophagy in pericytes. Autophagy plays a dual role in pericytes, especially under diabetic conditions, by influencing pericyte migration and survival. Dysregulated autophagy can lead to pericyte dysfunction, affecting BBB integrity and contributing to vascular complications in diabetes.

The original study published in this Research Topic by Huang et al. investigates the mechanisms of liver fibrosis and identifies a novel function of anthrax toxin receptor 2 (ANTXR2) in the liver. Hepatocellular carcinoma (HCC) also known as liver cancer, occurs when a tumor grows within the liver. HCC death rates are rising around the world, especially in Western countries. Liver fibrosis remains the highest risk factor for HCC, affecting blood circulation in the liver. Interestingly, due to lifestyle changes, alcoholism has contributed more than hepatitis B or C infections to liver fibrosis. By analyzing single-cell RNA sequencing data from cirrhotic and healthy livers, this group found that ANTXR2, expressed in ECs, plays a crucial role in regulating matrix metalloproteinase 2 (MMP2) activity. MMP2 degrades the extracellular matrix (ECM) and prevents excessive fibrosis. Knockout experiments showed that the absence of ANTXR2 in ECs led to more severe liver fibrosis, while overexpression reduced it. The protective role of ANTXR2 in liver fibrosis makes it a promising therapeutic target.

Another interesting study, a data report by Gao et al., sheds light on how ECs play a vital role in vascular health, and their senescence contributes to aging and disease. This group has studied the effects of telomerase reverse transcriptase (TERT) gene knockout (KO) in EC and focused on its telomere-independent functions. TERT protects against senescence, and its inactivation in ECs leads to increased expression of senescence markers and endothelial dysfunction, especially in adipose tissue. Using RNA sequencing and single-cell analysis, the study shows that TERT-EC-KO mice fed a high-calorie diet exhibit enhanced cellular aging and immune cell changes. The findings highlight the role of TERT in EC health, providing a model to study endothelial aging and potential therapies.

Lastly, the Xu et al. study explores the connection between two vascular disorders: proliferative diabetic retinopathy (PDR) and Alzheimer’s disease (AD). PDR is serious complication of Diabetic Retinopathy (DR) where abnormal new blood vessels grow on the retina. DR is a vascular complication of diabetes that occurs when high blood sugar levels damage the blood vessels in the retina. Vascular changes also play a central role in the progression of the AD. This group has reanalyzed and compared the scRNA-seq data of human fibrovascular membrane (FVM) of PDR and sn-RNA seq data from human hippocampus vasculature of AD. They found that amyloid-beta precursor protein (APP) signaling pathway is responsible for the interactions in the vasculature between PDR and AD as it influences both ECs and macrophages. The findings suggest that DR may serve as a biomarker for AD risk, highlighting shared pathogenic pathways.

While the concepts related to vascular diseases have been recognized for centuries, significant milestones emerged in 20th century with the ability to identify and intervene vascular diseases with medications. The understanding of vascular diseases continues to evolve with advancements in medical research and technology.

## References

[B1] Peng H. L. Wang X. L. Du J. B. Cui Q. H. Huang Y. Q. Jin H. F. (2021). Metabolic reprogramming of vascular endothelial cells: basic research and clinical applications. Front. Cell Dev. Biol. 9. 10.3389/fcell.2021.626047 PMC7930387 33681205

[B2] Pober J. S. Min W. Bradley J. R. (2009). Mechanisms of endothelial dysfunction, injury, and death. Annu. Rev. Pathology-Mechanisms Dis. 4, 71–95. 10.1146/annurev.pathol.4.110807.092155 18754744

[B3] Ungvari Z. Tarantini S. Kiss T. Wren J. D. Giles C. B. Griffin C. T. (2018). Endothelial dysfunction and angiogenesis impairment in the ageing vasculature. Nat. Rev. Cardiol. 15 (9), 555–565. 10.1038/s41569-018-0030-z 29795441 PMC6612360

